# Improvement in risk prediction for patients with atrial fibrillation and intermediate-risk CHA_2_DS_2_-VASc score utilizing highly sensitive cardiac troponin T

**DOI:** 10.1371/journal.pone.0330164

**Published:** 2025-08-21

**Authors:** Christian Salbach, Mustafa Yildirim, Barbara Ruth Milles, Hauke Hund, Matthias Mueller-Hennessen, Norbert Frey, Hugo Katus, Evangelos Giannitsis

**Affiliations:** Department of Internal Medicine III, Cardiology, University Hospital of Heidelberg, Heidelberg, Germany; Wiltse Memorial Hospital, KOREA, REPUBLIC OF

## Abstract

**Background:**

Guidelines of the European Society of Cardiology recommend a clinical risk assessment for patients with atrial fibrillation (AF). However, scores such as the CHA_2_DS_2_-VASc score show only a modest performance for prediction of adverse endpoints.

**Methods:**

This retrospective single-center all-comer study uses data from the Heidelberg Registry of Atrial Fibrillation of 9,995 patients with non-valvular AF presenting to the emergency department (ED) of the University Hospital of Heidelberg from June 2009 until March 2020. Per CHA_2_DS_2_-VASc, risk was classified as low (0 point in men, ≤ 1 point in females), intermediate, or high (≥2 points in men and ≥3 points in females). The predictive performance of the CHA_2_DS_2_-VASc score, with and without highly sensitive cardiac troponin T (hs-cTnT), was evaluated for a composite endpoint comprising stroke, myocardial infarction (MI) or all-cause mortality.

**Results:**

Performance of the CHA_2_DS_2_-VASc score for the prediction of the composite endpoint was poor Area under the curve (AUC): 0.648 (95%CI: 0.638–0.657) particularly in patients at intermediate-risk AUC: 0.542 (95%CI: 0.508–0.575). Adding hs-cTnT improved discrimination substantially in intermediate-risk patients (AUC: 0.778, 95% CI: 0.748–0.805). Notably, no events occurred in intermediate-risk patients with undetectable hs-cTnT (<5 ng/L).

**Conclusion:**

In patients with AF at intermediate thromboembolic risk, the addition of hs-cTnT to the CHA₂DS₂-VASc score enhances prediction of adverse cardiovascular outcomes. Hs-cTnT may help identify patients who could benefit from anticoagulation, while also identifying a low-risk subgroup unlikely to experience events.

## Introduction

Patients with atrial fibrillation (AF) have a manifold higher risk of stroke and suffer from a substantial burden of the disease [1–3]. Recent improvements for stroke prevention in AF patients include the replacement of vitamin-K-antagonist (VKA) by direct oral anticoagulant (DOAC) agents such as thrombin or factor Xa-inhibitors [4]. In clinical reality, AF is the most frequent arrhythmia in the setting of an emergency department (ED), causing rising costs and health care burden [5,6]. For the reduction of stroke risk in AF patients, initiation of adequate anticoagulation has been shown to be valuable but must be carefully balanced against the risk of higher bleeding rates [7,8]. Thus, guidelines of the European Society of Cardiology (ESC) recommend a clinical risk-factor based scoring system for the prediction of a patient individual stroke and bleeding risk [7]. Herein, several scores exists including the CHA_2_DS_2_-, CHA_2_DS_2_-VA- and the CHA_2_DS_2_-VASc score for evaluating stroke risk and guide decision of oral anticoagulation. [7] Unfortunately the ability of the CHA_2_DS_2_-VASc score to predict stroke is modest which has prompted the introduction of other validated scores such as the ABC (age, biomarkers, clinical history) stroke risk score, ATRIA (Anticoagulation and Risk Factors in Atrial Fibrillation) score, ORBIT (Outcomes Registry for Better Informed Treatment of Atrial Fibrillation) score, and the GARFIELD-AF (Global Anticoagulant Registry in the FIELD – Atrial Fibrillation) tool [9–12]. Current guideline recommendations regarding initiation of oral anticoagulation are clear for patients at high and low risk, whereas decision-making and risk prediction remains uncertain for those at intermediate risk according to CHA₂DS₂-VASc or CHA₂DS₂-VA score. [7,13] No specific guideline recommendations definitively guides management in this subgroup, making individualized assessment particularly important. Given this clinical uncertainty, we focused on patients at intermediate risk according to CHA₂DS₂-VASc score to evaluate whether biomarker-based risk stratification could provide more precise risk stratification for adverse outcomes. Several studies have been prompted to improve risk prediction of risk-factor based scores by using biomarkers [14–18]. For Asian patients with clinically low-risk CHA_2_DS_2_-VASc score and non-valvular AF, current evidence suggests that biomarkers such as NT-proBNP and creatinine could refine stroke risk stratification and prediction of adverse outcomes [19]. Thus, the current study aimed to evaluate an improvement in risk prediction of the CHA_2_DS_2_-VASc score by using highly sensitive cardiac troponin T (hs-cTnT) and retrospective data from the Heidelberg Registry of Atrial Fibrillation (HERA-FIB).

## Materials and methods

### Study population, design and follow-up

**Fig 1:**
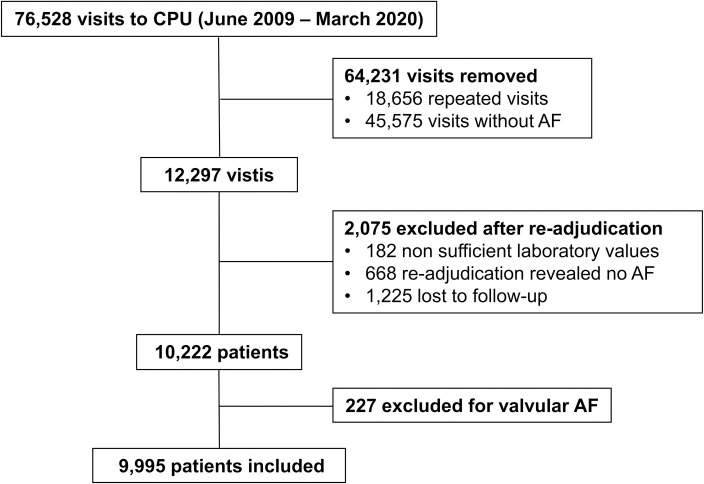
Flow-diagram for included AF patients admitted to the ED. The diagram shows patient recruitment after in- and exclusion of AF patients. Abbreviations: AF, atrial fibrillation; ED, emergency departement; hs-cTnT, highly sensitive cardiac troponin T.

HERA-FIB consecutively included AF patients presenting between June 2009 and March 2020 to the ED of University Hospital of Heidelberg. Design and rationale of HERA-FIB, such as inclusion and exclusion criteria were published earlier [20]. Inclusion criteria were comprehensive including age ≥ 18 years and AF either as primary reason for admission to the ED or as a comorbidity. Patients were excluded from the study for nonavailability of at least one hs-cTnT value or when lost to follow-up for all-cause mortality. For the current study, patients with valvular AF, defined as patients with mechanical prosthetic heart valve(s) or moderate/severe mitral stenosis were excluded [21]. A flow diagram for in- and excluded patients within this study is shown in **Fig 1**. For outcome parameters, a sequential follow-up method was performed as previously described including screening of electronical patient data, phone calls and postal questionnaires and at least a contact to registrational offices for vital status [20]. This study was conducted according to ethical principles stated in the Declaration of Helsinki (2008) and was reviewed and approved by the local ethics committee of the Medical Faculty of Heidelberg. Data was accessed for research purposes 1^st^ April 2024. No written informed consent was required. Patient identifiable data was pseudonymized to ensure data confidentiality and was not passed on to third parties. HERA-FIB is registered at ClinicalTrials.gov. Clinical Trials.gov identifier: NCT05995561.

### Definitions

For this study, a composite endpoint including stroke, myocardial infarction (MI) or all-cause mortality was chosen. Stroke events included ischemic or unknown cause of stroke but excluded hemorrhagic strokes, which were allocated as a major bleeding event according to International Society on Thrombosis and Hemostasis (ISTH) major bleeding criteria. The outcome of major bleeding events was defined according to ISTH major bleeding criteria. The CHA_2_DS_2_-VASc score was calculated retrospectively by adding two points for age ≥ 75 years and prior stroke, transient ischemic attack (TIA) or thromboembolism, as well as one point for congestive heart failure (defined by left ventricular ejection fraction <50%), arterial hypertension, age between 65–74 years, known diabetes mellitus, prior MI or peripheral artery disease, and female sex, respectively [7,22]. The CHA₂DS₂-VA score was calculated as described [13]. Risk categories comprised of a high risk category according to CHA₂DS₂-VASc (≥2 points in men, ≥ 3 points in women or CHA₂DS₂-VASc ≥ 2 points regardless of sex) an intermediate risk category (CHA₂DS₂-VASc = 1 point in men, 2 points in women or CHA₂DS₂-VASc = 1 point regardless of sex) and a low risk category (CHA₂DS₂-VASc = 0 points in men, ≤ 1 point in women, or CHA₂DS₂-VASc = 0 points regardless of sex). For calculation of the hs-cTnCHA_2_DS_2_VASc-score, the original CHA_2_DS_2_-VASc score was extended by adding 0 points for hs-cTnT < 5 ng/L, 1 point for hs-cTnT 5–14 ng/L and 2 points for hs-cTnT > 14 ng/L. Throughout the study period, hs-cTnT was measured using the Roche Cobas E411 platform (Roche Diagnostics Ltd., Rotkreuz, Switzerland), with no changes in vendor. Test kits were updated as per manufacturer recommendations over time, but the fundamental analytical platform remained unchanged.

### Statistical analysis

Data is presented as means (standard deviations, SD), medians (25^th^, 75^th^ percentiles, IQR), Kaplan-Meier estimates, as well as counts or percentages. For continuous variables the Kolmogorov-Smirnov test was used to test for normal distribution. Subgroups of categorical variables were tested against each other using chi-squared test or Fisher’s exact test. For continuous variables an unpaired Student’s t-test or Wilcoxon rank-sum test was performed. For Kaplan-Meier analysis, subgroups were compared by Log-rank test. The proportional hazards assumption was analyzed by the Grambsch and Therneau method [23]. We used time dependent receiver-operating-characteristic (ROC) curves and calculated the area under the ROC curves (AUC) on censored survival data. Here, the 95% confidence interval (CI) was calculated according to Hanley and McNeil [24]. For comparison of ROC curves, method by Hanley and McNeil was used as described [25]. The incremental prognostic value of adding hs-cTnT to the CHA₂DS₂-VASc score was assessed using the Net Reclassification Index (NRI), following established methodology for survival data [26]. A two tailed P-value of <0.05 was considered to indicate statistical significance. Statistical analyses were performed using R software (version 4.3.0, R Foundation for Statistical Computing, Vienna, Austria) as well as MedCalc (Version 20.105. MedCalc Software Ltd, Ostend Belgium).

## Results

In this study, 227 (2.2%) of initially 10,222 included AF patients within HERA-FIB were excluded for the presence of mechanical prosthetic heart valve(s) or moderate/severe mitral stenosis, resulting in a total of 9,995 included patients with non-valvular AF (**Fig 1**). Baseline characteristics are reported, stratified by clinical risk category of CHA_2_DS_2_-VASc score. A total of 455 patients (4.6%) were classified as low-risk, 871 patients (8.7%) as intermediate-risk and 8,669 patients (86.7%) as high-risk (**Table 1**). A figure for the distribution of the CHA_2_DS_2_-VASc score among the whole cohort is shown in S1 Fig. Patients subcategorized using CHA_2_DS_2_-VASc score differed significantly with respect to the baseline variables. The majority of the patients in the intermediate-risk group were male (65.7%), median age was 59 years (IQR 52–64). Within intermediate-risk group, the most frequent comorbidity was arterial hypertension (54.9%), followed by coronary artery disease (6.3%) and former MI (2.3%). A total of 61.1% of patients categorized intermediate-risk by CHA_2_DS_2_-VASc score received an oral anticoagulation (OAC) at discharge. Heart rate at admission was the highest in intermediate-risk category. Cardiac biomarkers such as hs-cTnT showed a steady increase among risk all risk categories.

**Table 1:** Baseline characteristics classified by CHA_2_DS_2_-VASc risk category.

Variables	Low-risk	Intermediate-risk	High-risk	p-value
Age, y, median (IQR)	51 (44-57)	59 (52-64)	77 (70-83)	<0.0001
Sex, male, n (%_all_)	316 (69.5)	572 (65.7)	4939 (57.0)	<0.0001
BMI, kg/m^²^, median (IQR)	25.8 (23.1-28.6) n = 219	26.5 (23.6-30.8) n = 494	26.8 (24.1-30.6) n = 5525	<0.0001
HF, bpm, median (IQR)	106 (82-135)	111 (85-135)	88 (72-114)	<0.0001
Bp_syst_, median (IQR)	140 (126-152) n = 450	145 (130-156) n = 863	147 (131-161) n = 8631	<0.0001
Bp_diast_, median (IQR)	85 (77-97) n = 450	89 (79-100) n = 863	84 (75-96) n = 8629	<0.0001
Arterial hypertension, n (%_all_)	0 (0)	478 (54.9)	7756 (89.5)	<0.0001
Diabetes mellitus, n (%_all_)	0 (0)	13 (1.5)	1983 (22.9)	<0.0001
History of CAD, n (%_all_)	0 (0)	55 (6.3)	4253 (29.1)	<0.0001
History of CABG, n (%_all_)	0 (0)	5 (0.6)	905 (10.4)	<0.0001
History of MI, n (%_all_)	0 (0)	20 (2.3)	1627 (18.8)	<0.0001
CHA_2_DS_2_-VASc -score, median (IQR)	0 (0-1)	1 (1-2)	4 (3-5)	<0.0001
OAC at discharge, n (%_all_)	200 (44.0)	532 (61.1)	6201 (71.5)	<0.0001
No OAC at discharge, n (%_all_)	255 (56.0)	339 (38.9)	2468 (28.5)	<0.0001
hs-cTnT, ng/L, median (IQR)	6 (4-10)	8 (6-15)	21 (12-41)	<0.0001
Hb, g/dl, median (IQR)	14.8 (13.8-15.7)	14.5 (13.4-15.5)	13.0 (11.6-14.3)	<0.0001
Serum creatinine, mg/dl, median (IQR)	0.85 (0.72-0.99)	0.89 (0.74-1.03)	1.01 (0.82-1.34)	<0.0001

Abbreviations: AF, atrial fibrillation; BMI, body mass index; bpm, beats per minutes; bp blood pressure; CABG, coronary artery bypass graft; CAD, coronary artery disease; dia, diastolic; Hb, hemoglobin; HF heart frequency; hs-cTnT, highly sensitive cardiac troponin T; MI, myocardial infarction; NT-proBNP, sys, systolic.

### Deterioration of adverse events in higher CHA_2_DS_2_-VASc -risk categories

**Fig 2:**
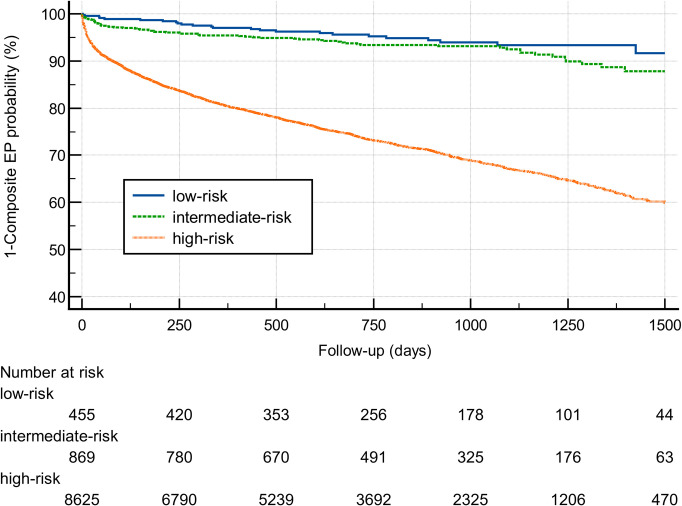
Kaplan Meier analysis for composite endpoint consisting of stroke, MI or all-cause mortality showed an increase in outcomes stratified by CHA_2_DS_2_-VASc risk category. Patients within higher risk categories of the CHA_2_DS_2_-VASc score showed a higher probability for the composite endpoint. Abbreviations: EP, endpoint.

Outcomes and hazard ratios (HR) classified by risk category of the CHA_2_DS_2_-VASc score are shown in **Table 2**. During a median follow-up of 23 months (IQR 12–35), the composite endpoint defined as stroke, MI or all-cause mortality event occurred in 5.3% for low-risk patients, 7.3% for intermediate-risk patients and 28.6% for patients within the high-risk category. A Kaplan-Meier analysis classified by CHA_2_DS_2_-VASc-risk category for the composite endpoint is shown in **Fig 2**. Here, patients within the intermediate-risk category showed an HR of 1.43 (95%CI: 1.16–1.76, p < 0.0001) for an adverse outcome compared to low-risk category and an HR of 4.53 (95%CI: 3.97–5.15, p < 0.0001) for an adverse outcome compared to high-risk category. Compared to patients with low-risk, patients within the intermediate CHA_2_DS_2_-VASc-risk category had an HR of 2.39 (95% CI: 1.28–4.47, p < 0.0001) for stroke and an HR of 3.73 (95%CI: 2.31–6.03, p < 0.0001) for a major bleeding event. Kaplan-Meier analyses for separate outcome parameters and other outcomes recorded within HERA-FIB, such as all-cause mortality, stroke, major bleeding and myocardial infarction (MI) stratified by CHA_2_DS_2_-VASc score risk category is shown in S2 Fig.

**Table 2:** Outcomes and HR classified by CHA_2_DS_2_-VASc risk category.

Variables	Low-risk	Intermediate-risk	High-risk	p-value
Composite EP^*^, n (%_**all**_)	24 (5.3)	64 (7.3)	2476 (28.6)	<0.0001
HR (95%CI)		1.43 (1.16-1.76)	6.45 (5.42-7.67)	<0.0001
**Stroke, n (%**_**all**_)	2 (0.5)	9 (1.2)	271 (3.7)	<0.0001
HR (95% CI)		2.39 (1.28-4.47)	8.50 (5.04-14.32)	<0.0001
**MI, n (%**_**all**_)	5 (1.2)	8 (1.0)	364 (5.0)	<0.0001
HR (95%CI)		0.85 (0.50-1.46)	4.67 (3.00-7.31)	<0.0001
**All-cause mortality, n (%**_**all**_)	18 (4.0)	49 (5.6)	2038 (23.5)	<0.0001
HR (95% CI)		1.45 (1.15-1.83)	6.88 (5.66-8.35)	<0.0001
**Major bleeding, n (%**_**all**_)	3 (0.7)	21 (2.7)	456 (6.2)	<0.0001
HR (95% CI)		3.73 (2.31-6.03)	9.53 (6.38-14.21)	<0.0001

* the composite EP consisted of stroke, MI or all-cause mortality, p-value was calculated as p-value for trend and log rank test for HRs. Abbreviations: CI, confidence interval; HR, hazard ratio; MI, myocardial infarction.

### Modest prognostic performance of CHA_2_DS_2_-VASc score

For the entire cohort, the AUC for the CHA_2_DS_2_-VASc score in prediction of the composite endpoint was 0.648 (95%CI: 0.638–0.657, p < 0.0001). For the individual components of the composite endpoint, CHA_2_DS_2_-VASc score showed only a modest performance with an AUC of 0.623 (95%CI: 0.612–0.633, p < 0.0001) for stroke, an AUC of 0.632 (95%CI: 0.621–0.642, p < 0.0001) for MI and an AUC of 0.643 (95%CI: 0.634–0.652, p < 0.0001) for all-cause mortality. For major bleeding events, AUC of the CHA_2_DS_2_-VASc score was 0.604 (95%CI: 0.594–0.615, p < 0.0001) for the entire cohort.

### Improved prediction of adverse outcomes adding hs-cTnT

**Fig 3:**
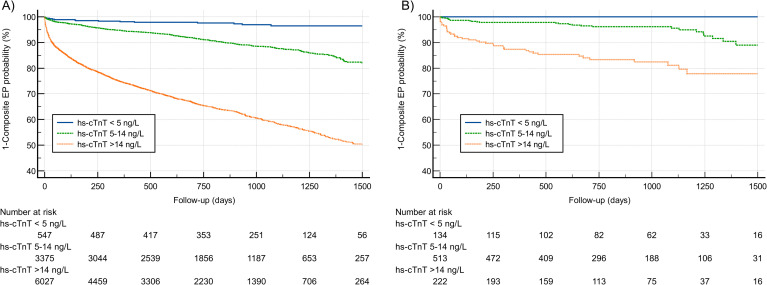
Kaplan Meier analysis for the composite endpoint stratified by hs-cTnT categories for the entire cohort (A) and patients in intermediate-risk category according to CHA_2_DS_2_-VASc score (B). Abbreviations: EP, endpoint; hs-cTnT, highly sensitive cardiac troponin T.

Since the CHA_2_DS_2_-VASc score showed only a modest performance in prediction of the composite endpoint, we thought to evaluate the role of a biomarker enhanced risk score utilizing hs-cTnT. For the prediction of the composite endpoint, hs-cTnT alone showed an AUC of 0.745 (95%CI: 0.737–0.754, p < 0.0001) within the entire study cohort. AUCs for hs-cTnT separated for the individual endpoints are shown in S1 Table. A Kaplan-Meier analysis for the composite endpoint and hs-cTnT categories is shown in **Fig 3A**. HRs for the composite endpoint increased among hs-cTnT categories. Compared to patients with hs-cTnT serum levels <5 ng/L, patients with a hs-cTnT serum level between 5–14 ng/L had a HR of 4.06 (95%CI: 3.45–4.79) for the composite endpoint. In patients with hs-cTnT serum level >14 ng/L, HR for the composite endpoint was 17.19 (95%CI: 14.65–20.18), respectively. Kaplan-Meier analyses separated for available outcomes classified by hs-cTnT levels (hs-cTnT < 5 ng/l, hs-cTnT 5–14 ng/L and hs-cTnT > 14 ng/L) are shown in S3 Fig. To quantify the incremental predictive value of hs-cTnT when added to the CHA₂DS₂-VASc score, we calculated the NRI, which was 0.83 (95% CI: 0.56–0.98; p = 0.0099), indicating substantial improvement in risk stratification for adverse outcomes.

### Improvement of risk prediction by adding hs-cTnT in intermediate-risk AF patients

In AF patients with intermediate-risk CHA_2_DS_2_-VASc, AUC of the CHA_2_DS_2_-VASc score in prediction of the composite endpoint was 0.542 (95%CI: 0.508–0.575, p = 0.2487). Instrumenting hs-cTnT serum levels for risk prediction in patients with intermediate CHA_2_DS_2_-VASc score showed a significantly higher AUC for the prediction of the composite endpoint of 0.778 (95%CI: 0.748–0.805), ∆AUC 0.236 (95%CI 0.146–0.325, p < 0.001). Kaplan Meier analysis for patients within the intermediate-risk CHA_2_DS_2_-VASc category, stratified by hs-cTnT categories (hs-cTnT < 5 ng/l, hs-cTnT 5–14 ng/L and hs-cTnT > 14 ng/L) is shown in Fig 3B. In patients with undetectable hs-cTnT serum levels, i.e., values below the limit of detection (LoD) <5 ng/L, no composite endpoint event occurred, whereas rates of the composite endpoint increased gradually from 4.87% in patients with hs-cTnT of 5–14 ng/L to 17.41% in those with hs-cTnT serum levels >14 ng/L. Accordingly, HR for patients with hs-cTnT serum levels of >14 ng/L compared to patients with hs-cTnT serum levels 5–14 ng/L was 3.92 (95%CI: 2.16–7.09, p < 0.0001). Consistently, the addition of hs-cTnT to the CHA₂DS₂-VASc score in intermediate-risk patients significantly improved risk classification, as reflected by a NRI of 0.24 (95% CI: 0.12–0.47; p < 0.0001).

### Prediction of the composite endpoint in patients with intermediate CHA_2_DS_2_-VASc score treated with oral anticoagulants

Among 871 patients within the intermediate CHA_2_DS_2_-VASc category, a total of 532 (61.1%) patients were treated with oral anticoagulants. In OAC treated patients, AUC CHA_2_DS_2_-VASc score in prediction of the composite endpoint was 0.515 (95%CI: 0.472–0.558, p = 0.7958). For stroke prediction AUC was 0.572 (95%CI: 0.526–0.617, p = 0.5974) and for the prediction of a major bleeding event AUC was 0.641 (95%CI: 0.596–0.684, p = 0.0782). However, when hs-cTnT was added, AUC improved to 0.755 (95%CI: 0.716–0.791), p < 0.0001 in prediction of the composite endpoint.

### Prediction of the composite endpoint in patients with intermediate CHA_2_DS_2_-VASc score not treated with oral anticoagulants

In 38.9% of AF patients not treated with oral anticoagulants and categorized as intermediate risk per CHA_2_DS_2_-VASc, AUC for the CHA_2_DS_2_-VASc score in prediction of the composite endpoint was 0.559 (95%CI: 0.504–0.613, p = 0.2076). However, when hs-cTnT was added, AUC significantly improved to 0.808 (95%CI: 0.62–0.848, p < 0.0001) for prediction of the composite endpoint.

### Comparison of CHA₂DS₂-VASc to CHA₂DS₂-VA in risk assessment of patients within the intermediate risk group

According to the CHA₂DS₂-VASc score, 871 patients were classified as intermediate risk (1 point in men, 2 points in women). However, reclassification using the CHA₂DS₂-VA score resulted in a different distribution of risk groups, with 564 patients (5.6%) classified as low risk, 967 patients (9.7%) as intermediate risk, and 8,464 patients (84.7%) as high risk within the HERA-FIB cohort. As a result, the distribution of outcome parameters across risk categories differed between the two scoring models, as detailed in S2 Table. For the entire cohort, the CHA₂DS₂-VA score showed limited discriminatory ability for predicting the composite endpoint (AUC: 0.650, 95% CI: 0.641–0.660, p < 0.0001). A summary of AUC values for all outcome parameters is provided in S3 Table. Among patients with an intermediate CHA₂DS₂-VA score, the predictive value for the composite endpoint was poor (AUC: 0.509, 95% CI: 0.475–0.542, p = 0.8185). However, when hs-cTnT was incorporated within patients with intermediate CHA₂DS₂-VA score, the predictive performance significantly improved, with an AUC of 0.778 (95% CI: 0.750–0.803, p < 0.0001). A Kaplan-Meier analysis for the composite endpoint in intermediate-risk patients per CHA₂DS₂-VA score is shown in S4 Fig. Among the 967 patients classified as intermediate risk by CHA₂DS₂-VA, 611 (63.2%) received OAC, while 356 (36.8%) remained without OAC treatment. For the prediction of the composite endpoint, the CHA₂DS₂-VA score alone showed poor discrimination in both OAC-treated patients (AUC: 0.500, 95% CI: 0.460–0.540) and non-OAC patients (AUC: 0.500, 95% CI: 0.447–0.553). However, the addition of hs-cTnT significantly improved risk prediction, showing an AUC of 0.733 (95% CI: 0.696–0.767, p < 0.0001) in OAC-treated patients and 0.825 (95% CI: 0.782–0.863, p < 0.0001) in non-OAC patients.

### Risk assessment using a novel hs-cTnCHA_2_DS_2_VASc-score

Considering that serum levels of hs-cTnT > 5 ng/L and hs-cTnT > 14 ng/L remained independent predictors for the composite endpoint in patients with intermediate-risk CHA_2_DS_2_-VASc score, we evaluated an extension of the original score by adding 0 points for hs-cTnT < 5 ng/L, 1 point for hs-cTnT 5–14 ng/L and 2 points for hs-cTnT > 14 ng/L. The median for hs-cTnCHA_2_DS_2_VASc-score in patients within the intermediate risk category was 2 points (IQR 2–3).

Importantly, no events were observed in intermediate risk patients with hs-cTnT < 5 ng/L, while the event rate increased to 5.0% in the 5–14 ng/L group and reached 17.5% in those with hs-cTnT > 14 ng/L, indicating a clear gradient of risk across hs-cTnT strata.

In all patients, a comparison between the original CHA_2_DS_2_VASc score and the hs-cTnCHA_2_DS_2_VASc-score in prediction of the composite endpoint revealed an AUC of 0.647 (95%CI 0.638–0.657) for the original CHA_2_DS_2_VASc-score and an AUC of 0.683 (95%CI 0.674–0.692) for the hs-cTnCHA_2_DS_2_VASc-score, ∆AUC 0.0358 (95%CI 0.0325–0.0390, p < 0.0001). In patients with oral anticoagulants and an intermediate CHA_2_DS_2_-VASc score, AUC for hs-cTnCHA2DS2VASc-score in prediction of the composite endpoint was 0.683 (95%CI: 0.642–0.723) showing a ∆AUC of 0.168 (95%CI: 0.0767–0.260, p = 0.0003) in comparison to the original CHA_2_DS_2_-VASc score. In intermediate risk patients without oral anticoagulants AUC for hs-cTnCHA2DS2VASc-score in prediction of the composite endpoint was 0.686 (95%CI: 0.631–0.732, ∆AUC in comparison to the original CHA_2_DS_2_-VASc score was 0.124 (95%CI: 0.0367–0.212, p = 0.0054).

## Discussion

This study sought to investigate whether hs-cTnT might improve prediction of a composite endpoint including stroke, MI and all-cause mortality in AF patients at an intermediate-risk according to the CHA_2_DS_2_-VASc score. The later has only modest performance for prediction of the composite endpoint and does not help for the decision whether AF patients at intermediate risk should receive an anticoagulation. Our findings show that the addition of hs-cTnT to the CHA_2_DS_2_-VASc score improves the prediction of the composite endpoint in AF patients presenting in the setting of an ED. A unique finding of our study is that patients with undetectable hs-cTnT represent a very low risk cohort in respective to stroke, MI or all-cause mortality.

In order to identify appropriate candidates for oral anticoagulation, guidelines on management of AF recommend a clinical risk-factor based assessment for prediction of stroke and major bleeding events [7]. However, scoring systems recommended by guidelines show only a modest performance and fail to provide clear recommendations on important subgroups of patients, such as patients with intermediate-risk CHA_2_DS_2_-VASc score (men: 1 point, women: 2 points) [7,22,27]. Beyond its use in AF-related thromboembolic risk stratification, emerging evidence highlights that the CHA₂DS₂-VASc score has broader prognostic utility in cardiovascular conditions. Studies have demonstrated its predictive value for major adverse cardiovascular events (MACE) in patients with coronary artery disease (CAD), acute coronary syndrome (ACS), and even in individuals without AF. For example, Hioki et al. [28] showed that the CHA₂DS₂-VASc score effectively stratifies risk in patients undergoing percutaneous coronary intervention (PCI), while Sen et al. [29] found its association with cardiovascular mortality and MACE in ACS patients. Furthermore, it has been applied to non-thromboembolic cardiovascular risks, including mortality in hospitalized cardiovascular patients and even pneumonia-related cardiovascular events. Lee et al. [30] demonstrated its relevance in predicting hospital readmission rates among patients receiving tele-health monitoring, while Wang et al. [31] showed that it stratifies MACE risk in pneumonia patients. Additionally, Cheng et al. [32] validated its prognostic role in PAD patients undergoing angioplasty.

The 2024 ESC Guidelines now recommend the use of the CHA₂DS₂-VA score instead of CHA₂DS₂-VASc for thromboembolic risk stratification in AF patients [13]. However, the role of sex as a risk factor in anticoagulation decision-making remains controversial. Large population-based studies have yielded conflicting results regarding the predictive superiority of CHA₂DS₂-VA over CHA₂DS₂-VASc [33]. For example, an analysis of UK primary and secondary care data from 195,719 AF patients found that removing female sex from the CHA₂DS₂-VASc score did not reduce its ability to discriminate thromboembolic events, challenging the necessity of this adjustment [34]. In our cohort, we compared both risk scores in patients classified as intermediate risk. We found that the predictive ability of CHA₂DS₂-VA for stroke, MI and all-cause mortality remained poor in patients at intermediate risk (AUC: 0.509, 95% CI: 0.475–0.542 p = 1.00), similar to CHA₂DS₂-VASc. However, when hs-cTnT was incorporated, predictive performance significantly improved for both scores, irrespective of oral anticoagulation. Specifically, the AUC for the composite endpoint increased to 0.778 (95% CI: 0.750–0.803, p < 0.0001). The improvement was observed both in patients receiving OAC and in those not treated with anticoagulation, with a more pronounced effect in non-OAC patients. These findings highlight the limitations of both CHA₂DS₂-VASc and CHA₂DS₂-VA in risk stratification for intermediate-risk AF patients and suggest that biomarker-enhanced models such as hs-cTnT could improve risk assessment in clinical routine. Given that guideline recommendations remain uncertain in this subgroup, the integration of hs-cTnT may help individualize anticoagulation strategies in patients who otherwise fall into a risk “gray zone”. Our results reveal that patients classified as intermediate-risk based on the CHA_2_DS_2_-VASc score had a significantly higher HR for adverse outcomes when compared to low-risk patients. Limitations of the CHA_2_DS_2_-VASc score prevail across all risk categories but individualization of therapy has the highest clinical relevance among patients at intermediate risk, where clear recommendations for the initiation of anticoagulation are missing. Here, we could show a more accurate discrimination of risk assessment by integrating hs-cTnT serum levels in risk prediction for stroke, MI and all-cause mortality. The significant increase in HR, as hs-cTnT levels rose indicates its potential as a valuable biomarker for refining risk stratification in AF patients. The most relevant finding is that patients with undetectable hs-cTnT represent a cohort at very low risk experiencing no adverse outcome events of stroke, MI or all-cause mortality during follow-up. In fact, within the intermediate-risk group, no events occurred in patients with hs-cTnT < 5 ng/L, whereas the event rate for the composite outcome (stroke, MI, and all-cause mortality) increased to 5.0% in those with hs-cTnT between 5–14 ng/L and reached 17.5% in those with hs-cTnT > 14 ng/L, showing a clear, step wise increase in adverse outcomes. These findings suggest that intermediate-risk patients with hs-cTnT < 5 ng/L represent a cohort of extremely low risk for adverse events. The present study contributes to the continuous evolution of risk stratification in AF patients presenting to an emergency department. Historically, stroke risk prediction models, including CHA₂DS₂-VASc, were developed in cohorts of untreated AF patients. However, in contemporary practice, stroke risk in AF represents a mixture of residual risk despite OAC and the true risk in untreated patients. As Lip et al. (2023) emphasize, interpreting stroke risk in modern AF cohorts must account for the impact of anticoagulation use. [35] In our study, 61% of intermediate-risk patients received OAC, while 39% remained untreated, reflecting this dual-risk profile. Thus, even when separated by OAC or non-OAC therapy, the addition of hs-cTnT to the CHA_2_DS_2_-VASc score added relevant predictive information for the composite endpoint of stroke, MI and all-cause mortality

Given the broad cardiovascular implications of the CHA₂DS₂-VASc score, the use of a composite endpoint consisting of stroke, MI and all-cause mortality our study is justified. Previous studies have shown that ischemic and hemorrhagic risks are closely interlinked, as seen in patients with CAD, heart failure, and peripheral artery disease. Thus, incorporating both stroke and major bleeding into the risk assessment provides a more holistic approach to patient management.

The reasons why hs-cTn allows prediction of adverse outcomes in AF patients is not fully understood but most likely multifactorial as cardiac troponin summarizes the cumulative impact of comorbidities that trigger myocardial injury. In agreement with previous observations bleeding risk is closely linked to ischemic risk [36–39]. A subanalysis from the RE-LY study (Randomized Evaluation of Long-Term Anticoagulation Therapy), which was initially designed to investigate the appropriateness of dabigatran in comparison of warfarin, investigated the role of elevated cardiac troponin I in a preselected cohort of patients with new onset AF and found that cTnI serum levels were indepenently related to stroke [40]. However, the authors could not provide data on cTnT. Moreover, the RE-LY trial did not provide information for patients in real-world and particularly in the setting of admission to an ED. The hypothesis that biomarkers might improve prediction of adverse outcomes including stroke and adverse outcomes is not novel. Previously, Hijazi et al. introduced and validated the ABC score, a risk assessment tool that incorporates clinical information and biomarkers [9]. While prediction of adverse outcomes was improved, the implementation into clinical routine lags behind expectations outside randomized trials. The combination of the established CHA_2_DS_2_VASc-score with the single broadly available hs-cTnT or hs-cTnI which is used as a dichotomous variable is more convenient and more likely to be adopted in routine. However, future external validation of this combined risk assessment strategy is required before our findings will help to individualize anticoagulation in AF patients at intermediate stroke risk.

Our study introduces the hs-cTnCHA_2_DS_2_VASc-score as a novel predictive tool for assessing the combined endpoint of stroke, MI and all-cause mortality in AF patients with intermediate-risk in CHA_2_DS_2_-VASc score. Despite being widely used in clinical routine, our study underscores the modest performance of CHA_2_DS_2_-VASc score emphasizing the need for innovative and simple approaches to improve risk prediction in AF patients presenting in the acute setting of an ED. Particularly, the enhanced predictive value of hs-cTnT aligns with previous research suggesting that biomarkers – especially cardiac biomarkers – as demonstrated by studies [14–16,26,41], may significantly contribute to risk stratification for AF patients. Notably, the inclusion of hs-cTnT significantly enhances the discriminatory power of the risk assessment tool, within the intermediate-risk CHA_2_DS_2_-VASc-category. Beyond improvements in discrimination, we also evaluated reclassification performance. For the whole cohort, adding hs-cTnT to the CHA₂DS₂-VASc score resulted in an NRI of 0.83 (95% CI: 0.56–0.98; p = 0.0099), indicating substantial improvement in risk categorization. Among intermediate-risk patients, the NRI was 0.24 (95% CI: 0.12–0.47; p < 0.0001), reflecting clinically meaningful reclassification within this “gray-zone” population. This underscores the clinical value of hs-cTnT in refining risk assessment for AF patients, particularly in the intermediate-risk group, potentially offering a more precise basis for decisions regarding anticoagulation therapy. Moreover, given the increasing use of CHA₂DS₂-VASc in cardiovascular risk assessment beyond AF, our study provides further insights into how this score can be adapted for broader clinical applications.

## Limitations and future directions

It is important to acknowledge the limitations of our study. First, our findings were derived from a single tertiary referral center at an academic center in Germany and may not be generalized to other geographic regions or health care systems. Second, our findings on the predictive role of hs-cTnT alone and particularly the novel hs-cTnCHA_2_DS_2_VASc-score still requires prospective validation in independent populations. Third, our findings are based exclusively on hs-cTnT and no blood specimen are available for evaluation of other hs-cTnI assays. Fourth, the relatively short median follow-up duration of 23 months may not fully capture the long-term effects of risk assessment using the hs-cTnCHA_2_DS_2_VASc-score. Fifth, we studied the added value of hs-cTnT when combined with the CHA_2_DS_2_-VASc score but did not evaluate other risk prediction models such as the ABC score which already contains biomarkers. Finally, temporal changes in patient management and healthcare practices have changed over the study period, spanning from June 2009 to March 2020. In particular, we cannot exclude that changes in demographic characteristics such as age, as well as the increasing preference of DOAC versus VKA may have an impact on risk of stroke and other outcomes in contemporary clinical practice. Within HERA-FIB, the majority of patients were stratified as high risk per CHA₂DS₂-VASc. However, the current analysis focused exclusively on patients with intermediate risk, as they represent the subgroup where clinical decision-making regarding anticoagulation therapy and risk stratification remains most uncertain. While our findings show that hs-cTnT improves risk stratification in this population, an important area for future research will be the refinement of risk assessment strategies in high-risk patients. Additional studies should evaluate whether biomarker-based models, such as the hs-cTnT-enhanced CHA₂DS₂-VASc score, could provide further prognostic discrimination within the high-risk category and identify subgroups with particularly elevated or lower risk within this population.

## Conclusions

In conclusion, in patients with AF and intermediate thromboembolic risk, the decision to initiate oral anticoagulation remains uncertain due to the modest predictive performance of current risk scores. Our study shows that adding hs-cTnT to the CHA₂DS₂-VASc score substantially improves risk stratification for adverse cardiovascular outcomes, including stroke, myocardial infarction, and all-cause mortality, particularly within this intermediate-risk population. Importantly, no events occurred in intermediate-risk patients with hs-cTnT levels below the limit of detection, whereas the event rate increased stepwise in patients with elevated hs-cTnT. The addition of hs-cTnT yielded a significant net reclassification improvement in this group, supporting its potential role in risk stratification. In conclusion, our study shows a strong value of hs-cTnT when added to the CHA₂DS₂-VASc score These findings highlight the promise of a biomarker-enhanced CHA₂DS₂-VASc score for individualized risk assessment in AF patients at intermediate risk. However, prospective validation in independent cohorts is needed before clinical implementation.

## Supporting information

S1 Fig Distribution of CHA_2_DS_2_-VASc score across the entire cohort. (TIF)

S2 Fig Kaplan-Meier analysis for all-cause mortality, stroke, major bleeding and myocardial infarction classified by CHA_2_DS_2_-VASc score risk category. Patients with a high-risk category according to CHA_2_DS_2_VASc-score had a higher all-cause mortality (A), a higher probability of ischemic stroke rate (B), a higher probability of major bleeding events (C) and higher risk for myocardial infarction (D). Abbreviatons: MI, myocardial infarction.
(TIF)

S3 Fig Kaplan-Meier analysis for all-cause mortality, stroke, major bleeding and myocardial infarction classified by hs-cTnT category. Patients with a high hs-cTnT had a higher all-cause mortality (A), a higher probability of ischemic stroke (B), a higher probability of major bleeding (C) and higher risk for myocardial infarction (D). Abbreviations: MI, myocardial infarction, hs-cTnT, highly sensitive cardiac troponin T.
(TIF)

S4 Fig Kaplan Meier analysis for the composite endpoint stratified by hs-cTnT categories for patients in intermediate-risk category according to CHA_2_DS_2_-VA score. Abbreviations: EP, endpoint; hs-cTnT, highly sensitive cardiac troponin T.
(TIF)

S1 Table AUC analysis for hs-cTnT in predicting outcome variables. Abbreviations: AUC, area under the curve, CI, confidence interval. (DOCX)

S2 Table Outcomes and HR classified by CHA₂DS₂-VA risk category. *The composite EP consisted of stroke or major bleeding, p-value was calculated as p-value for trend and log rank test for HRs. Abbreviations: CI, confidence interval; HR, hazard ratio; MI, myocardial infarction.
(DOCX)

S3 Table AUCs of the CHA_2_DS_2_VA score separated for outcome variables. *The composite EP consisted of stroke and major bleeding events. Abbreviations: AUC, area under the curve, CI, confidence interval, EP endpoint.
(DOCX)

## References

[1] Wolf PA, Abbott RD, Kannel WB. Atrial fibrillation as an independent risk factor for stroke: the Framingham Study. Stroke. 1991;22(8):983–8. doi: 10.1161/01.str.22.8.983 1866765

[2] Benjamin EJ, Muntner P, Alonso A, Bittencourt MS, Callaway CW, Carson AP, et al. Heart Disease and Stroke Statistics-2019 Update: A Report From the American Heart Association. Circulation. 2019;139(10):e56–528. doi: 10.1161/CIR.0000000000000659 30700139

[3] Hylek EM, Go AS, Chang Y, Jensvold NG, Henault LE, Selby JV, et al. Effect of intensity of oral anticoagulation on stroke severity and mortality in atrial fibrillation. N Engl J Med. 2003;349(11):1019–26. doi: 10.1056/NEJMoa022913 12968085

[4] Bode W, Ptaszek LM. Management of Atrial Fibrillation in the Emergency Department. Curr Cardiol Rep. 2021;23(12):179. doi: 10.1007/s11886-021-01611-2 34657210

[5] Deshmukh A, Iglesias M, Khanna R, Beaulieu T. Healthcare utilization and costs associated with a diagnosis of incident atrial fibrillation. Heart Rhythm O2. 2022;3(5):577–86. doi: 10.1016/j.hroo.2022.07.010 36340482 PMC9626881

[6] Milman B, Burns BD. Atrial fibrillation: an approach to diagnosis and management in the emergency department. Emerg Med Pract. 2021;23(5):1–28. 33885254

[7] Hindricks G, Potpara T, Dagres N, Arbelo E, Bax JJ, Blomström-Lundqvist C, et al. 2020 ESC Guidelines for the diagnosis and management of atrial fibrillation developed in collaboration with the European Association for Cardio-Thoracic Surgery (EACTS): The Task Force for the diagnosis and management of atrial fibrillation of the European Society of Cardiology (ESC) Developed with the special contribution of the European Heart Rhythm Association (EHRA) of the ESC. Eur Heart J. 2021;42(5):373–498. doi: 10.1093/eurheartj/ehaa612 32860505

[8] Gorog DA, Gue YX, Chao T-F, Fauchier L, Ferreiro JL, Huber K, et al. Assessment and mitigation of bleeding risk in atrial fibrillation and venous thromboembolism: A Position Paper from the ESC Working Group on Thrombosis, in collaboration with the European Heart Rhythm Association, the Association for Acute CardioVascular Care and the Asia-Pacific Heart Rhythm Society. Europace. 2022;24(11):1844–71. doi: 10.1093/europace/euac020 35323922 PMC11636575

[9] Hijazi Z, Oldgren J, Lindbäck J, Alexander JH, Connolly SJ, Eikelboom JW, et al. The novel biomarker-based ABC (age, biomarkers, clinical history)-bleeding risk score for patients with atrial fibrillation: a derivation and validation study. Lancet. 2016;387(10035):2302–11. doi: 10.1016/S0140-6736(16)00741-8 27056738

[10] Fang MC, Go AS, Chang Y, Borowsky LH, Pomernacki NK, Udaltsova N, et al. A new risk scheme to predict warfarin-associated hemorrhage: The ATRIA (Anticoagulation and Risk Factors in Atrial Fibrillation) Study. J Am Coll Cardiol. 2011;58(4):395–401. doi: 10.1016/j.jacc.2011.03.031 21757117 PMC3175766

[11] O’Brien EC, Simon DN, Thomas LE, Hylek EM, Gersh BJ, Ansell JE, et al. The ORBIT bleeding score: a simple bedside score to assess bleeding risk in atrial fibrillation. Eur Heart J. 2015;36(46):3258–64. doi: 10.1093/eurheartj/ehv476 26424865 PMC4670965

[12] Fox KAA, Lucas JE, Pieper KS, Bassand J-P, Camm AJ, Fitzmaurice DA, et al. Improved risk stratification of patients with atrial fibrillation: an integrated GARFIELD-AF tool for the prediction of mortality, stroke and bleed in patients with and without anticoagulation. BMJ Open. 2017;7(12):e017157. doi: 10.1136/bmjopen-2017-017157 29273652 PMC5778339

[13] Van Gelder IC, Rienstra M, Bunting KV, Casado-Arroyo R, Caso V, Crijns HJGM, et al. 2024 ESC Guidelines for the management of atrial fibrillation developed in collaboration with the European Association for Cardio-Thoracic Surgery (EACTS). Eur Heart J. 2024;45(36):3314–414. doi: 10.1093/eurheartj/ehae176 39210723

[14] Berg DD, Ruff CT, Jarolim P, Giugliano RP, Nordio F, Lanz HJ, et al. Performance of the ABC Scores for Assessing the Risk of Stroke or Systemic Embolism and Bleeding in Patients With Atrial Fibrillation in ENGAGE AF-TIMI 48. Circulation. 2019;139(6):760–71. doi: 10.1161/CIRCULATIONAHA.118.038312 30586727 PMC6363338

[15] Berg DD, Ruff CT, Morrow DA. Biomarkers for Risk Assessment in Atrial Fibrillation. Clin Chem. 2021;67(1):87–95. doi: 10.1093/clinchem/hvaa298 33313695

[16] Hijazi Z, Oldgren J, Siegbahn A, Wallentin L. Application of Biomarkers for Risk Stratification in Patients with Atrial Fibrillation. Clin Chem. 2017;63(1):152–64. doi: 10.1373/clinchem.2016.255182 27811208

[17] Yildirim M, Salbach C, Reich C, Milles BR, Biener M, Frey N, et al. Comparison of the clinical chemistry score to other biomarker algorithms for rapid rule-out of acute myocardial infarction and risk stratification in patients with suspected acute coronary syndrome. Int J Cardiol. 2024;400:131815. doi: 10.1016/j.ijcard.2024.131815 38278492

[18] Yildirim M, Reich C, Salbach C, Biener M, Mueller-Hennessen M, Sörensen NA, et al. Identification of patients with suspected NSTE-ACS in the observe zone: evaluating GRACE 1.0 score and a biomarker panel for risk stratification and management optimization. Clin Res Cardiol. 2025;114(6):783–95. doi: 10.1007/s00392-025-02642-3 40227426 PMC12089253

[19] Shin SY, Han S-J, Kim J-S, Im SI, Shim J, Ahn J, et al. Identification of Markers Associated With Development of Stroke in “Clinically Low-Risk” Atrial Fibrillation Patients. J Am Heart Assoc. 2019;8(21):e012697. doi: 10.1161/JAHA.119.012697 31668140 PMC6898804

[20] Salbach C, Yildirim M, Hund H, Biener M, Mueller-Hennessen M, Frey N, et al. Design, rationale and initial findings from the Heidelberg Registry of Atrial Fibrillation on 10,222 patients with atrial fibrillation presenting to an emergency department over an 11-year period. Manuscript submitted for publication 2024. 10.1161/JAHA.123.033396 PMC11179873 38639359

[21] Lip GYH, Collet JP, Caterina R de, Fauchier L, Lane DA, Larsen TB, et al. Antithrombotic therapy in atrial fibrillation associated with valvular heart disease: a joint consensus document from the European Heart Rhythm Association (EHRA) and European Society of Cardiology Working Group on Thrombosis, endorsed by the ESC Working Group on Valvular Heart Disease, Cardiac Arrhythmia Society of Southern Africa (CASSA), Heart Rhythm Society (HRS), Asia Pacific Heart Rhythm Society (APHRS), South African Heart (SA Heart) Association and Sociedad Latinoamericana de Estimulación Cardíaca y Electrofisiología (SOLEACE). Europace. 2017;19(11):1757–8. doi: 10.1093/europace/eux240 29096024

[22] Lip GYH, Nieuwlaat R, Pisters R, Lane DA, Crijns HJGM. Refining clinical risk stratification for predicting stroke and thromboembolism in atrial fibrillation using a novel risk factor-based approach: the euro heart survey on atrial fibrillation. Chest. 2010;137(2):263–72. doi: 10.1378/chest.09-1584 19762550

[23] Grambsch PM, Therneau TM, Fleming TR. Diagnostic plots to reveal functional form for covariates in multiplicative intensity models. Biometrics. 1995;51(4):1469–82. 8589234

[24] Hanley JA, McNeil BJ. The meaning and use of the area under a receiver operating characteristic (ROC) curve. Radiology. 1982;143(1):29–36. doi: 10.1148/radiology.143.1.7063747 7063747

[25] Hanley JA, McNeil BJ. A method of comparing the areas under receiver operating characteristic curves derived from the same cases. Radiology. 1983;148(3):839–43. doi: 10.1148/radiology.148.3.6878708 6878708

[26] Pencina MJ, D’Agostino RB Sr, D’Agostino RB, Vasan RS. Evaluating the added predictive ability of a new marker: from area under the ROC curve to reclassification and beyond. Stat Med. 2008;27(2):157–72; discussion 207-12. doi: 10.1002/sim.2929 17569110

[27] Chen J-Y, Zhang A-D, Lu H-Y, Guo J, Wang F-F, Li Z-C. CHADS2 versus CHA2DS2-VASc score in assessing the stroke and thromboembolism risk stratification in patients with atrial fibrillation: a systematic review and meta-analysis. J Geriatr Cardiol. 2013;10(3):258–66. doi: 10.3969/j.issn.1671-5411.2013.03.004 24133514 PMC3796700

[28] Hioki H, Miura T, Miyashita Y, Motoki H, Shimada K, Kobayashi M, et al. Risk stratification using the CHA2DS2-VASc score in patients with coronary heart disease undergoing percutaneous coronary intervention; sub-analysis of SHINANO registry. Int J Cardiol Heart Vasc. 2015;7:76–81. doi: 10.1016/j.ijcha.2015.02.007 28785649 PMC5497243

[29] Sen J, Tonkin A, Varigos J, Fonguh S, Berkowitz SD, Yusuf S, et al. CHA2DS2-VASc and CHADS2 scores for risk stratification of major adverse cardiovascular events in the COMPASS trial. European Heart Journal. 2020;41(Supplement_2). doi: 10.1093/ehjci/ehaa946.2906

[30] Lee J-K, Hung C-S, Huang C-C, Chen Y-H, Chuang P-Y, Yu J-Y, et al. Use of the CHA2DS2-VASc Score for Risk Stratification of Hospital Admissions Among Patients With Cardiovascular Diseases Receiving a Fourth-Generation Synchronous Telehealth Program: Retrospective Cohort Study. J Med Internet Res. 2019;21(1):e12790. doi: 10.2196/12790 30702437 PMC6374726

[31] Wang B-Y, Lin F-Y, Ku M-S, Wang Y-H, Lee K-Y, Ho S-W. CHA2DS2-VASc Score for Major Adverse Cardiovascular Events Stratification in Patients with Pneumonia with and without Atrial Fibrillation. J Clin Med. 2021;10(18):4093. doi: 10.3390/jcm10184093 34575202 PMC8466520

[32] Cheng Y-T, Chang F-L, Li P-H, Lu W-C, Chiu C-S. Assessing the Suitability of CHA2DS2-VASc for Predicting Adverse Limb Events and Cardiovascular Outcomes in Peripheral Artery Disease Patients with Percutaneous Transluminal Angioplasty: A Retrospective Cohort Study. Biomedicines. 2024;12(6):1374. doi: 10.3390/biomedicines12061374 38927581 PMC11202305

[33] Champsi A, Mobley AR, Subramanian A, Nirantharakumar K, Wang X, Shukla D, et al. Gender and contemporary risk of adverse events in atrial fibrillation. Eur Heart J. 2024;45(36):3707–17. doi: 10.1093/eurheartj/ehae539 39217497 PMC11439109

[34] Yoshimura H, Providencia R, Finan C, Schmidt AF, Lip GYH. Refining the CHA2DS2VASc risk stratification scheme: shall we drop the sex category criterion?. Europace. 2024;26(11):euae280. doi: 10.1093/europace/euae280 39522169 PMC11574618

[35] Lip GYH, Proietti M, Potpara T, Mansour M, Savelieva I, Tse HF, et al. Atrial fibrillation and stroke prevention: 25 years of research at EP Europace journal. Europace. 2023;25(9):euad226. doi: 10.1093/europace/euad226 37622590 PMC10451006

[36] Yeh RW, Secemsky EA, Kereiakes DJ, Normand S-LT, Gershlick AH, Cohen DJ, et al. Development and Validation of a Prediction Rule for Benefit and Harm of Dual Antiplatelet Therapy Beyond 1 Year After Percutaneous Coronary Intervention. JAMA. 2016;315(16):1735–49. doi: 10.1001/jama.2016.3775 27022822 PMC5408574

[37] Costa F, van Klaveren D, James S, Heg D, Räber L, Feres F, et al. Derivation and validation of the predicting bleeding complications in patients undergoing stent implantation and subsequent dual antiplatelet therapy (PRECISE-DAPT) score: a pooled analysis of individual-patient datasets from clinical trials. Lancet. 2017;389(10073):1025–34. doi: 10.1016/S0140-6736(17)30397-5 28290994

[38] Ueki Y, Bär S, Losdat S, Otsuka T, Zanchin C, Zanchin T, et al. Validation of the Academic Research Consortium for High Bleeding Risk (ARC-HBR) criteria in patients undergoing percutaneous coronary intervention and comparison with contemporary bleeding risk scores. EuroIntervention. 2020;16(5):371–9. doi: 10.4244/EIJ-D-20-00052 32065586

[39] Brabrand M, Lassen AT, Knudsen T, Hallas J. Seven-day mortality can be predicted in medical patients by blood pressure, age, respiratory rate, loss of independence, and peripheral oxygen saturation (the PARIS score): a prospective cohort study with external validation. PLoS One. 2015;10(4):e0122480. doi: 10.1371/journal.pone.0122480 25867881 PMC4395094

[40] Hijazi Z, Oldgren J, Andersson U, Connolly SJ, Ezekowitz MD, Hohnloser SH, et al. Cardiac biomarkers are associated with an increased risk of stroke and death in patients with atrial fibrillation: a Randomized Evaluation of Long-term Anticoagulation Therapy (RE-LY) substudy. Circulation. 2012;125(13):1605–16. doi: 10.1161/CIRCULATIONAHA.111.038729 22374183

[41] Providência R, Paiva L, Barra S. Risk stratification of patients with atrial fibrillation: Biomarkers and other future perspectives. World J Cardiol. 2012;4(6):195–200. doi: 10.4330/wjc.v4.i6.195 22761972 PMC3386309

