# Supplementary figures and images for: Improvement in risk prediction for patients with atrial fibrillation and intermediate-risk CHA2DS2-VASc score utilizing highly sensitive cardiac troponin T
Source: PLoS One. 2025 Aug 21;20(8):e0330164. doi: 10.1371/journal.pone.0330164 (PMC12370022; doi:10.1371/journal.pone.0330164)

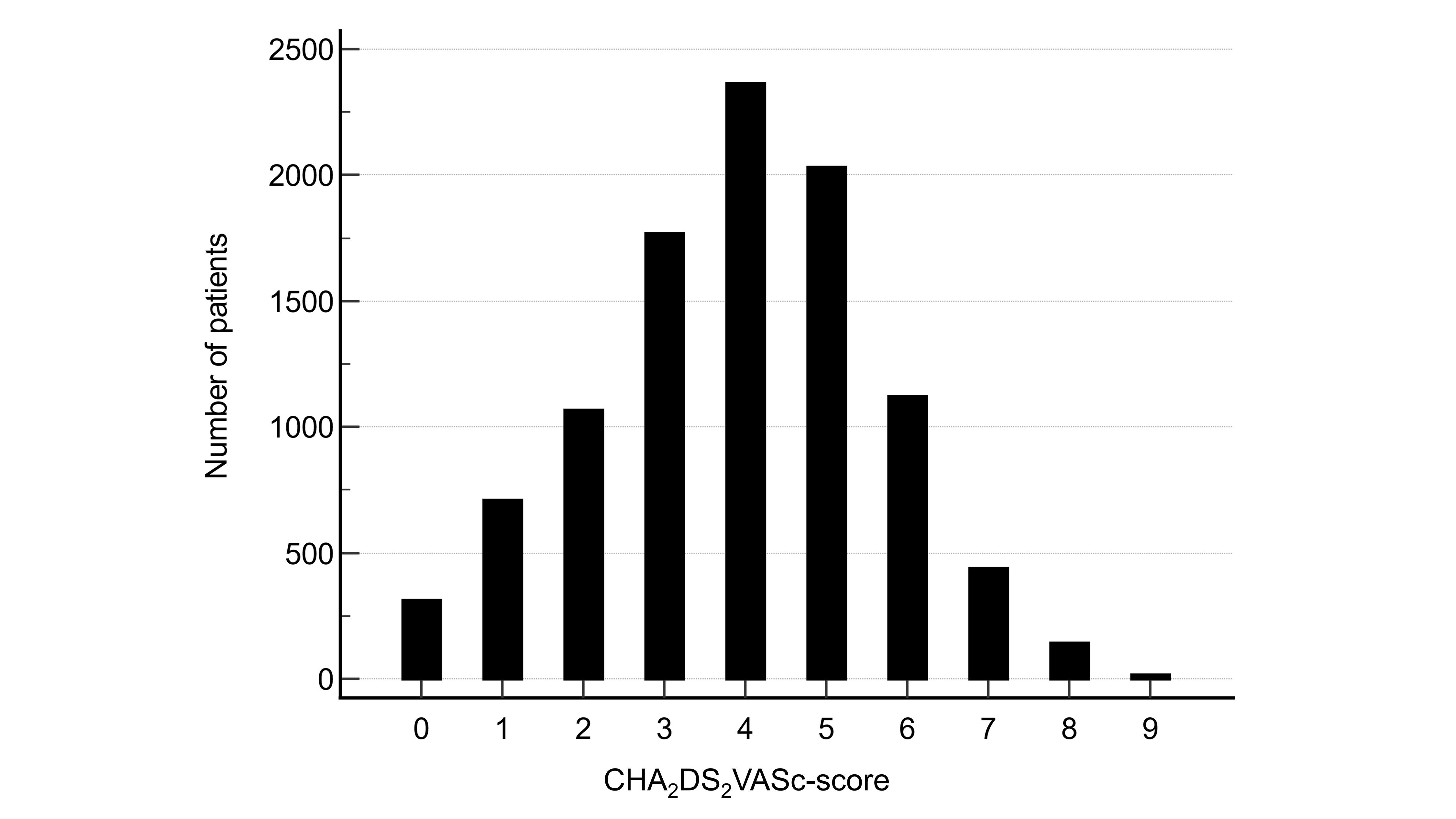

Supplement: S1 Fig — (TIF) [file pone.0330164.s001.tif]

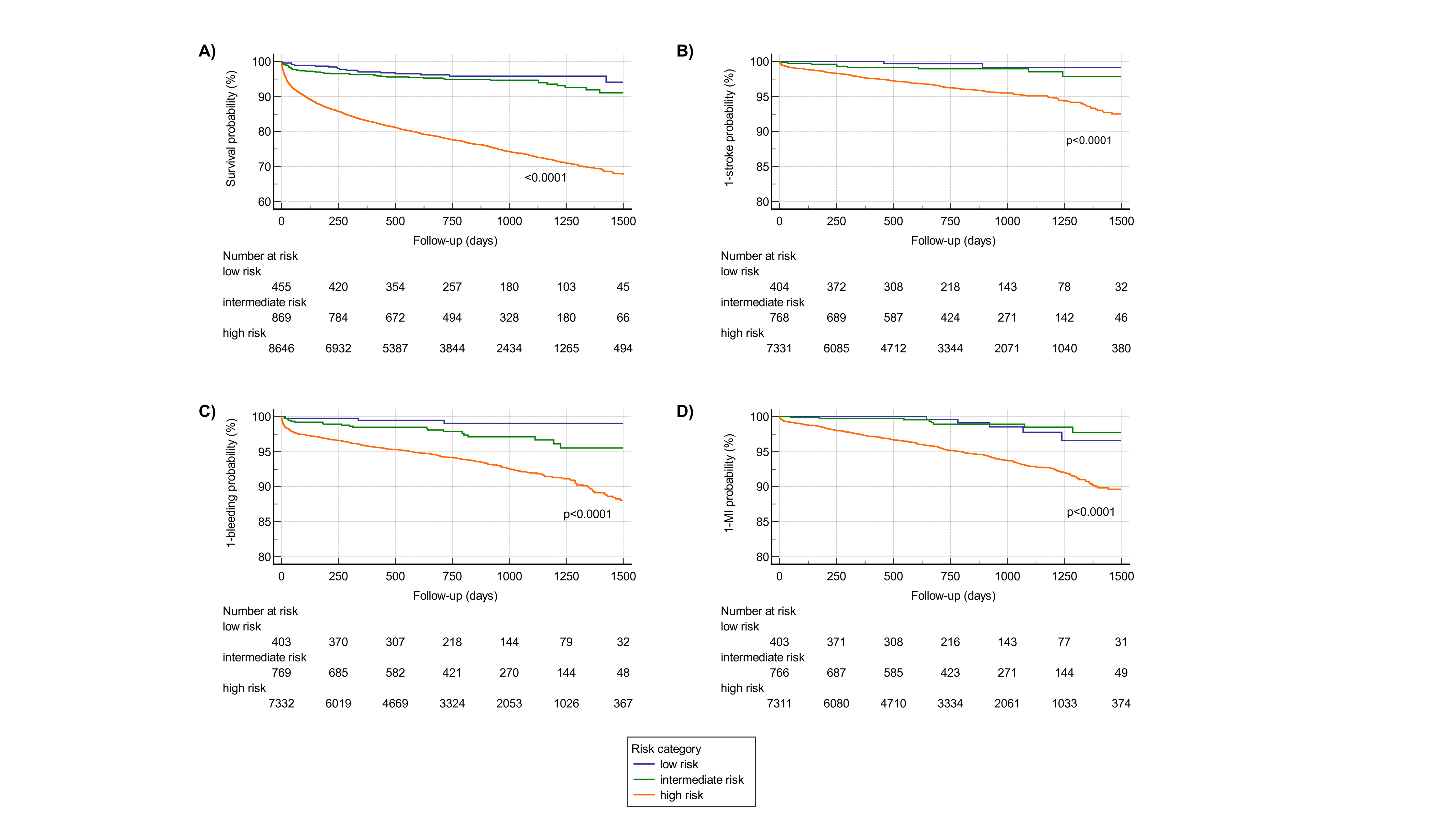

Supplement: S2 Fig — Patients with a high-risk category according to CHA2DS2VASc-score had a higher all-cause mortality (A), a higher probability of ischemic stroke rate (B), a higher probability of major bleeding events (C) and higher risk for myocardial infarction (D). Abbreviatons: MI, myocardial infarction. (TIF) [file pone.0330164.s002.tif]

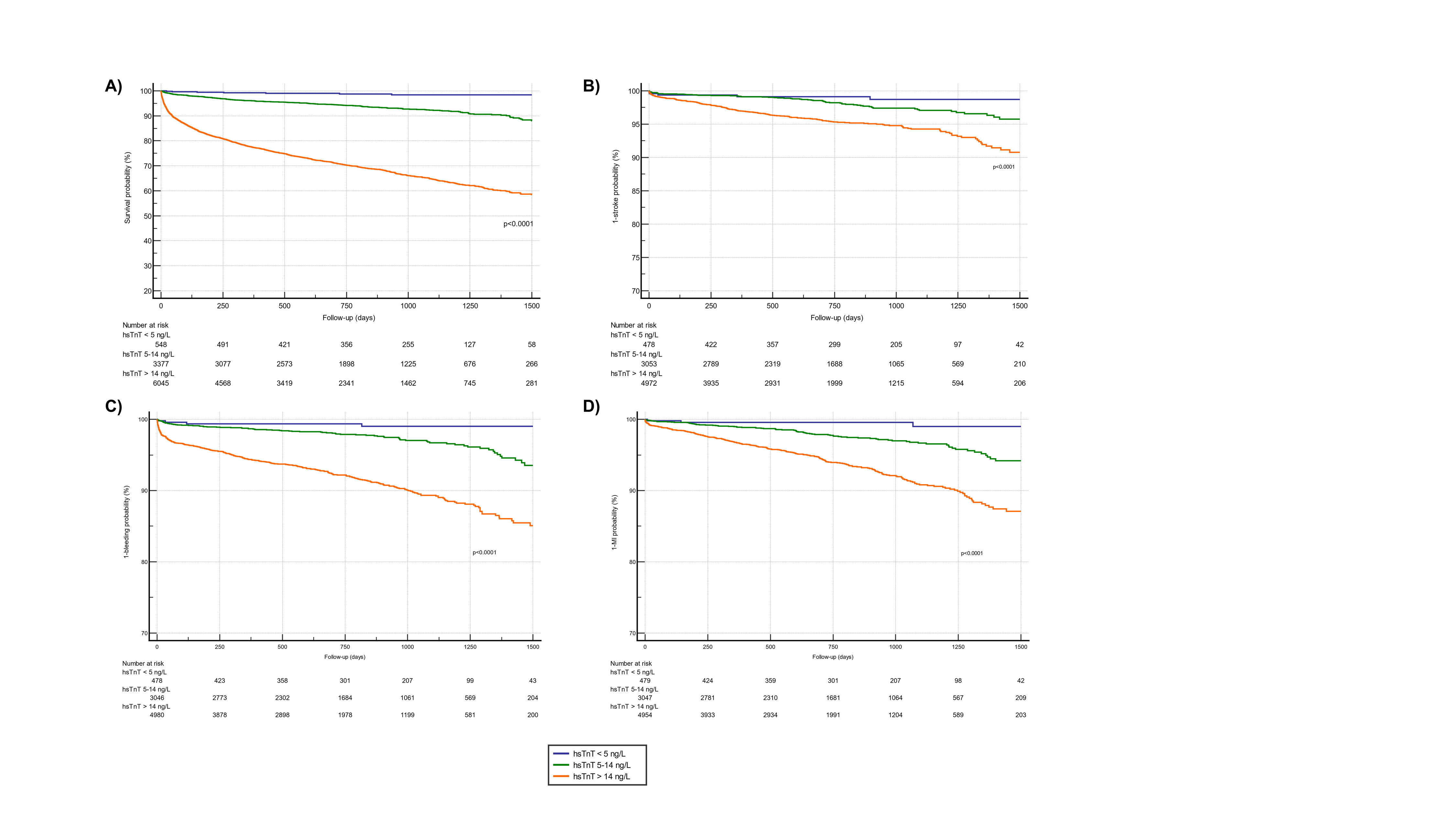

Supplement: S3 Fig — Patients with a high hs-cTnT had a higher all-cause mortality (A), a higher probability of ischemic stroke (B), a higher probability of major bleeding (C) and higher risk for myocardial infarction (D). Abbreviations: MI, myocardial infarction, hs-cTnT, highly sensitive cardiac troponin T. (TIF) [file pone.0330164.s003.tif]

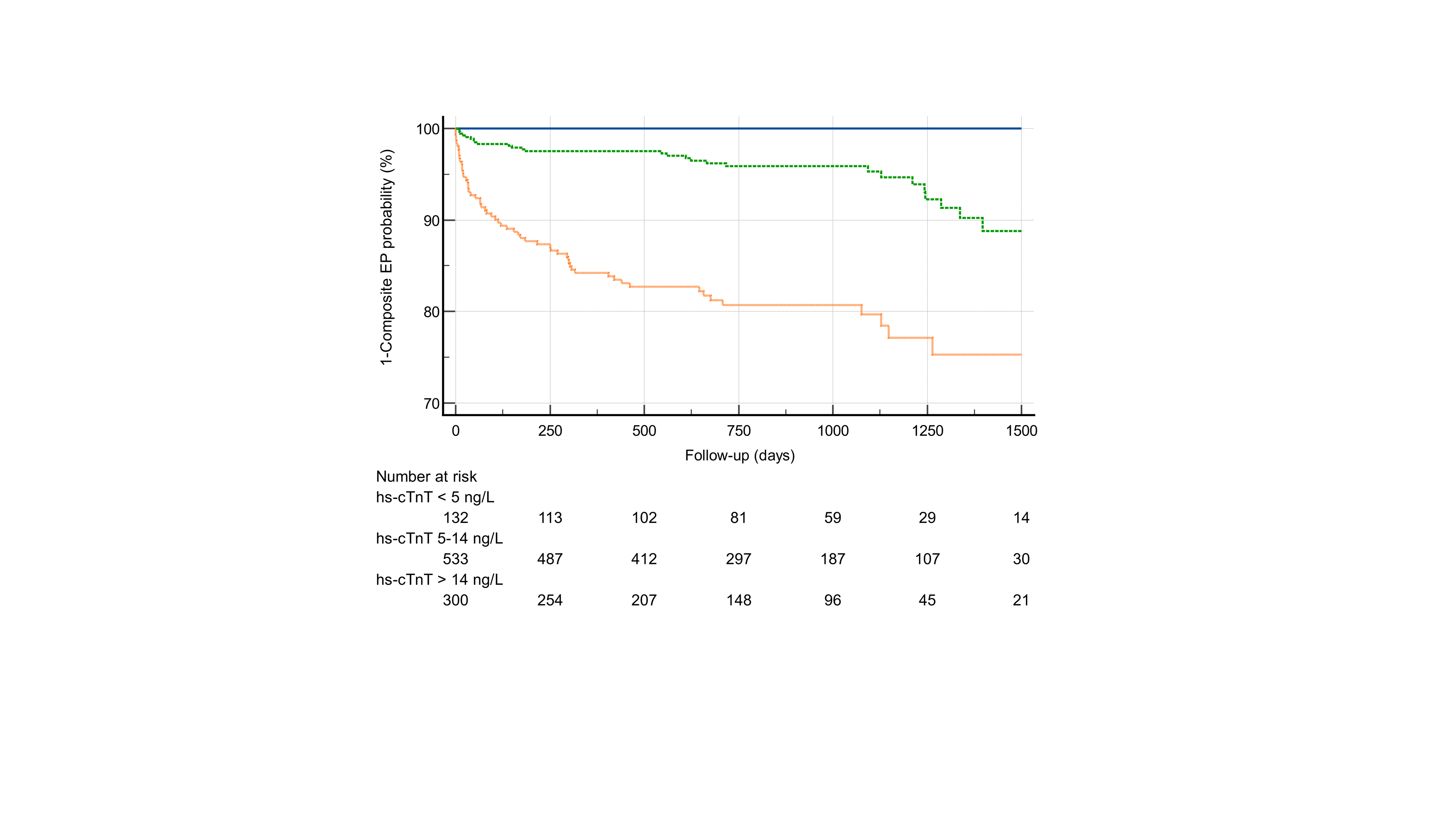

Supplement: S4 Fig — Abbreviations: EP, endpoint; hs-cTnT, highly sensitive cardiac troponin T. (TIF) [file pone.0330164.s004.tif]
