# Supplementary material for: Improvement in risk prediction for patients with atrial fibrillation and intermediate-risk CHA2DS2-VASc score utilizing highly sensitive cardiac troponin T
Source: PLoS One. 2025 Aug 21;20(8):e0330164. doi: 10.1371/journal.pone.0330164 (PMC12370022; doi:10.1371/journal.pone.0330164)
Supplement: S1 Table — (DOCX) [file pone.0330164.s005.docx]

**S1 Table. AUCs for hs-cTnT in predicting the separate outcome variables.**

| **Outcome variable** | **AUC (95% CI)** |
| --- | --- |
| Stroke | 0.603 (0.593 - 0.613) |
| Major bleeding | 0.652 (0.642 - 0.662) |
| All-cause mortality | 0.763 (0.755 - 0.771) |
| Myocardial infarction | 0.656 (0.646 - 0.666) |

Abbreviations: AUC, area under the curve, CI, confidence interval.
