# Supplementary material for: Improvement in risk prediction for patients with atrial fibrillation and intermediate-risk CHA2DS2-VASc score utilizing highly sensitive cardiac troponin T
Source: PLoS One. 2025 Aug 21;20(8):e0330164. doi: 10.1371/journal.pone.0330164 (PMC12370022; doi:10.1371/journal.pone.0330164)
Supplement: S2 Table — *The composite EP consisted of stroke or major bleeding, p-value was calculated as p-value for trend and log rank test for HRs. Abbreviations: CI, confidence interval; HR, hazard ratio; MI, myocardial infarction. (DOCX) [file pone.0330164.s006.docx]

**S3 Table. Outcomes and HR classified by CHA₂DS₂-VA risk category**

| **Variables** | **Low-risk** | **Intermediate-risk** | **High-risk** | **p-value** |
| --- | --- | --- | --- | --- |
| **Composite EP*, n (%_all_)** | 31 (5.5) | 86 (8.9) | 2447 (28.9) | <0.0001 |
| HR (95%CI) |  | 1.67 (1.38-2.03) | 6.20 (5.30-7.26) | <0.0001 |
| **Stroke, n (%_all_)** | 3 (0.6) | 12 (1.4) | 267 (3.7) | <0.0001 |
| HR (95% CI) |  | 2.36 (1.32-4.20) | 7.01 (4.36-11.28) | <0.0001 |
| **All-cause mortality, n (%_all_)** | 25 (4.4) | 67 (6.9) | 2013 (23.8) | <0.0001 |
| HR (95% CI) |  | 1.61 (1.30-1.99) | 6.15 (5.16-7.33) | <0.0001 |
| **MI, n (%_all_)** | 5 (1.0) | 11 (1.27) | 361 (5.1) | <0.0001 |
| HR (95%CI) |  | 1.30 (0.79-2.13) | 5.81 (3.86-8.73) | <0.0001 |
| **Major bleeding, n (%_all_)** | 6 (1.2) | 25 (2.9) | 449 (6.3) | <0.0001 |
| HR (95% CI) |  | 3.73 (2.31-6.03) | 9.53 (6.38-14.21) | <0.0001 |

*the composite EP consisted of stroke, MI or all-cause mortality. P-value was calculated as p-value for trend and log rank test for HRs. Abbreviations: CI, confidence interval; HR, hazard ratio; MI, myocardial infarction.
