# Supplementary material for: Improvement in risk prediction for patients with atrial fibrillation and intermediate-risk CHA2DS2-VASc score utilizing highly sensitive cardiac troponin T
Source: PLoS One. 2025 Aug 21;20(8):e0330164. doi: 10.1371/journal.pone.0330164 (PMC12370022; doi:10.1371/journal.pone.0330164)
Supplement: S3 Table — *The composite EP consisted of stroke and major bleeding events. Abbreviations: AUC, area under the curve, CI, confidence interval, EP endpoint. (DOCX) [file pone.0330164.s007.docx]

**S2 Table. AUCs for CHA₂DS₂-VA score in predicting the separate outcome variables.**

| **Outcome variable** | **AUC (95% CI)** |
| --- | --- |
| Stroke | 0.623 (0.612 - 0.633) |
| Major bleeding | 0.600 (0.590 - 0.611) |
| All-cause mortality | 0.643 (0.634 - 0.653) |
| Myocardial infarction | 0.645 (0.635 - 0.655 |

Abbreviations: AUC, area under the curve, CI, confidence interval.
